# *In vitro *studies on the modification of low-dose hyper-radiosensitivity in prostate cancer cells by incubation with genistein and estradiol

**DOI:** 10.1186/1748-717X-3-19

**Published:** 2008-07-14

**Authors:** Robert Michael Hermann, Hendrik Andreas Wolff, Hubertus Jarry, Paul Thelen, Carsten Gruendker, Margret Rave-Fraenk, Heinz Schmidberger, Hans Christiansen

**Affiliations:** 1 Department of Radiotherapy and Radiooncology, University hospital Goettingen, Robert-Koch-Str. 40, 37075, Goettingen, Germany; 2 Department of Experimental Endocrinology, University hospital Goettingen, Robert-Koch-Str. 40, 37075, Goettingen, Germany; 3 Department of Urology, University hospital Goettingen, Robert-Koch-Str. 40, 37075, Goettingen, Germany; 4 Department of Gynecology, University hospital Göttingen, Robert-Koch-Str. 40, 37075, Göttingen, Germany; 5 Department of Radiotherapy, University of Mainz, Langenbeckstr, 1, 55131, Mainz, Germany

## Abstract

**Background:**

As the majority of prostate cancers (PC) express estrogen receptors, we evaluated the combination of radiation and estrogenic stimulation (estrogen and genistein) on the radiosensitivity of PC cells in vitro.

**Methods:**

PC cells LNCaP (androgen-sensitive) and PC-3 (androgen-independent) were evaluated. Estrogen receptor (ER) expression was analyzed by means of immunostaining. Cells were incubated in FCS-free media with genistein 10 μM and estradiol 10 μM 24 h before irradiation and up to 24 h after irradiation. Clonogenic survival, cell cycle changes, and expression of p21 were assessed.

**Results:**

LNCaP expressed both ER-α and ER-β, PC-3 did not. Incubation of LNCaP and PC-3 with genistein resulted in a significant reduction of clonogenic survival. Incubation with estradiol exhibited in low concentrations (0.01 μM) stimulatory effects, while higher concentrations did not influence survival. Both genistein 10 μM and estradiol 10 μM increased low-dose hyper-radiosensitivity [HRS] in LNCaP, while hormonal incubation abolished HRS in PC-3. In LNCaP cells hormonal stimulation inhibited p21 induction after irradiation with 4 Gy. In PC-3 cells, the proportion of cells in G2/M was increased after irradiation with 4 Gy.

**Conclusion:**

We found an increased HRS to low irradiation doses after incubation with estradiol or genistein in ER-α and ER-β positive LNCaP cells. This is of high clinical interest, as this tumor model reflects a locally advanced, androgen dependent PC. In contrast, in ER-α and ER-β negative PC-3 cells we observed an abolishing of the HRS to low irradiation doses by hormonal stimulation. The effects of both tested compounds on survival were ER and p53 independent. Since genistein and estradiol effects in both cell lines were comparable, neither ER- nor p53-expression seemed to play a role in the linked signalling. Nevertheless both compounds targeted the same molecular switch. To identify the underlying molecular mechanisms, further studies are needed.

## Background

Curative therapy of prostate carcinoma (PC) is of major concern, as PC is the leading cancer diagnosis in the male population [1]. In locally advanced tumor stages the recommended treatment is radiotherapy combined with simultaneous application of LHRH-agonists. [2].

Several studies reported that the majority of PC express estrogen receptors (ER-α and/or ER-β) [3-5]. The soy isoflavone genistein is a well-known ER-agonist. In contrast to estradiol it activates especially ER-β [6]. Therefore both substances exhibit distinct effects, and genistein containing soy products seem to have fewer side effects than estradiol in patients [7]. As estradiol (e.g. diethylstilbestrol) is associated with a high risk of cardiovascular side effects in patients, we compared the effects of estradiol with the better tolerable genistein in irradiated PC cell lines *in vitro*.

Other mechanisms for genistein action besides the ER mediated effects have been reported. It has been shown that genistein acts as an inhibitor of steroidogenesis, blocks several protein tyrosine- and histidine kinases [8-10], and inhibits topoisomerase I and II [11]. These effects result in alterations of several intracellular and extracellular pathways including cell cycle control, apoptosis, and angiogenesis [12-14].

In PC cell lines genistein incubation proved to have many effects *in vitro*. Among others, it inhibits proliferation [15], reduces PSA secretion [16] and induces dose-dependent apoptosis [17]. *In vivo*, soy extracts let to significant reduction in tumour progression on mice after subcutaneous implantation of PC cell lines [18].

The combination of radiotherapy and estrogenic stimulation can increase cytotoxicity [19]. This has been shown in particular for breast cancer cells [20]. Recently several studies have reported an enhancement of radiosensitivity by genistein in different tumor cell lines *in vitro*: in human esophageal squamous cell cancer cell lines (TP 53 mutant and wild-type) [21], hepatoma cells [22], leukemia cells [23], and PC cell lines [24,25]. Furthermore, increased radiosensitivity in the androgen independent PC cell line PC-3 has been demonstrated *in vitro *and *in vivo *[26,27].

Our study analyzes the interactions of irradiation and genistein or estradiol incubation in androgen sensitive LNCaP and androgen independant PC-3 cells *in vitro*. Clinically relevant irradiation doses between 0 and 4 Gy were tested.

## Methods

### Cell lines and cultures

PC cell lines LNCaP and PC-3 were purchased from DSMZ (Braunschweig, Germany). All cells were cultured in Dulbecco's minimal essential medium (phenol red free, high glucose [4,5 g/l]) supplemented with 2% glutamine, 1% sodium pyruvate (Sigma, Taufkirchen., Germany), 1% penicillin and streptomycin (Biochrom, Berlin, Germany) and 10% fetal bovine serum (PAA, Cölbe, Germany) in 10% CO2 atmosphere. The cells were grown as a monolayer culture, harvested and replated twice per week (PC-3) or once per week (LNCaP). To avoid genetic alterations in late cell passages, early passages were regularly taken from frozen stocks.

### Hormonal treatment and irradiation

Genistein and estradiol were purchased from Sigma. Both were dissolved in ethanol stock solution. To exclude any other than the studied hormonal effects, 24 h before genistein or estradiol were added the cell cultures were washed with PBS and supplemented with medium without FCS ("serum withdrawal").

**LNCaP **cells showed a long doubling time (about 5 days). Defined cell numbers were plated in 25 cm^2 ^tissue flasks. After attachment of the cells (about 48 h later) serum withdrawal was done, the next day genistein or estradiol in different concentrations and ethanol in the highest used concentration for the controls were added to the medium to incubate for another 24 h. Radiation was given with a linear accelerator (Varian, Palo Alto, USA) with 6 MeV and a dose rate of 2.4 Gy/min. 24 h later the medium was changed and the cells were incubated in medium supplemented with FCS.

As **PC-3 **cells had a short doubling time (about 1 day), irradiation experiments were performed as „immediate plating“. PC-3 cells were seeded in 25 cm^2 ^tissue culture flasks in 5 ml medium. After growing to 80% confluence, serum was withdrawn. 24 h later genistein or estradiol in different concentrations and ethanol in the highest used concentration for the controls were added to the medium to incubate for another 24 h. Immediately after irradiation, cells were trypsinized and counted. Serial dilution allowed to plate between 300 – 1000 cells in four new culture flasks in FCS supplemented medium.

### Colony forming assay

The cell survival was evaluated using a standard colony-forming assay. A total of 300 – 1000 cells were plated per 25 cm^2 ^flask for low to high doses of radiation. After more than 5 doublings the experiments were stopped. The cell layer was fixed with 70% ethanol and stained with crystal violet. Scoring was done under a microscope. Colonies with more than 50 cells were counted as survivors.

Each experiment was performed at least 3 times; each survival point was calculated from at least 12 single results. Cell survival was calculated as follows:

$$\text{S}=\frac{\text{no}\text{. of colonies counted at a given dose}}{\text{no}\text{. of cells plated at a given dose}}\times \frac{\text{control no}\text{. of cells plated}}{\text{control no}\text{. of colonies counted}}.$$

### Staining of ER-α and ER-β

Antibodies were purchased from Novocastra (Newcastle, UK). The protocols for immunostaining have been published previously [28]. In short, 10.000 cells of the cell lines were seeded in each well of an 8-chamber slide. 24 h later the cells were fixed with methanol and H_2_O_2_. After incubation with blocking solution, primary monoclonal mouse antibodies were given for 1 h (to stain for ER-α: NCL-ER-6F11 [Novocastra, Newcastle, UK] 1:80; for ER-β: NCL-ER- β [Novocastra] 1:200). After washing, the secondary anti-mouse antibody was incubated for 30 min. The plates were washed and stained with DAB (Sigma). To serve as positive and negative controls EFO-21 and BG-1 ovarian cancer cell lines were used.

### Protein extraction and Western Blot analysis of p21

Cells were grown to 80% confluence in 25 cm^2 ^culture flasks. After serum withdrawal for 24 h the cells were incubated with genistein and estradiol in different concentrations. 24 h later the culture flasks were irradiated with 0 Gy, 0.5 Gy and 4 Gy (linear accelerator, Varian). Protein extraction and Western Blots have been published elsewhere [28]. In short, 6 h later the cells were trypsinized washed and incubated with 200 μl 1 mM PMSF in PBS on ice. The probes were frozen three times in liquid nitrogen, and then centrifuged at 10.000 × g for 30 min. The protein concentration was measured in the supernatant using the DC protein assay kit (Bio-Rad, Hercules, USA) following the recommendations of the manufacturer. Protein aliquots (50 μg) were separated by size on a 10% SDS resolving gel and transferred to a nitrocellulose membrane. For protein detection the Western Breeze Chromogenic Immunodetection system (Invitrogen, Carlsbad, USA) was used following the instructions of the manufacturer. Primary antibodies were (all mice) for WAF-1 (Ab-1): monoclonal mouse IgG (Oncogene); and for actin IgG1 (Santa Cruz, Santa Cruz, USA). Incubation time of these antibodies was 90 min in a dilution of 1:1000.

### FACS analysis of cell cycle distribution

500.000 cells were seeded in 25 cm^2 ^flasks. After attaching and growing to 80% confluence, FCS was withdrawn. The next day hormones in different concentrations were added, after 24 h of incubation they were irradiated. During the whole process and at different time intervals after irradiation samples were washed twice with PBS, trypsinized, washed again and fixed with cold ethanol and stored at -18°C. After washing off ethanol, the cells were stained in 1 ml DAPI – solution (Partec, Muenster, Germany) and analyzed for cell cycle distribution in a flow cytometer (Partec).

### Statistical analysis

All experiments were repeated three times. For descriptive statistics, the software package KaleidaGraph 3.5 (Synergy Software, Reading, USA) was used. Means and standard deviations were calculated for each of the data points; statistical comparison of the survival data was done using the t-test and one-way ANOVA (Tukey HSD for post hoc testing). P < 0.05 was considered statistically significant. Survival curves, each referring to its specific control, were fitted to the data using the linear-quadratic model if possible (S = exp(-aD-βD^2^), S = surviving cells, D = radiation Dose, a,β = cell specific constants) [29].

## Results

### Receptor expression

**Figure 1:**
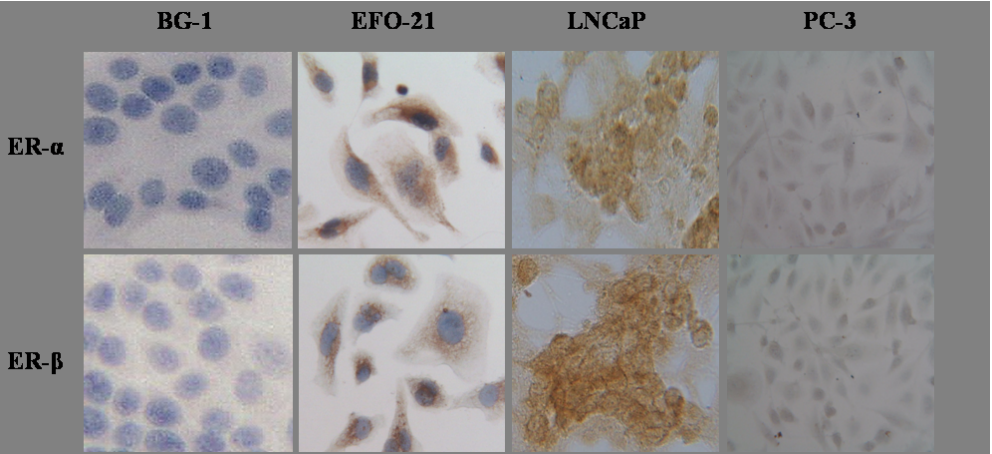
**Immuncytological staining of ER-α and -β in EFO-21 (positive control), BG-1 (negative control), LNCaP and PC-3**. In the first row ER-α has been stained, in the second ER-β. EFO-21 and BG-1 cells served as controls: EFO-21 is an ovarian carcinoma cell line that expresses both ER-α and ER-β, whereas BG-1 is an ovarian cell that does not express these receptors. Expression of the receptors reflects a brown staining. For easier analysis, staining of the nuclei with DAB was not performed in the presented samples of LNCaP and PC-3. While LNCaP cells showed expression of ER-α and ER-β, PC-3 cells did not.

Immunocytological staining for ER-α and ER-β revealed that LNCaP expressed both receptors (figure 1). In contrast, in our passages of PC-3 cells we could not stain any of these receptors.

### Genistein inhibits clonogenic cell survival in LNCaP and PC-3

In PC-3 cells we tested genistein concentrations between 0.1 μM and 25 μM, and estradiol concentrations between 0.01 μM and 10 μM. In LNCaP both hormones were used in concentrations between 0.01 μM and 10 μM.

**Figure 2:**
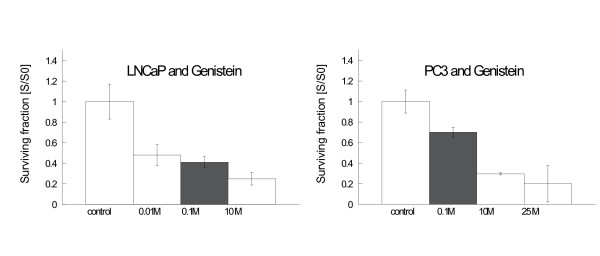
**Clonogenic survival LNCaP (left side) and PC-3 (right side) after incubation with genistein (LNCaP 48 h incubation, PC-3 24 h)**. Survival was expressed relative to untreated controls. Error bars represent standard errors. In both cell lines a significant reduction in colony forming is observed after incubation with genistein. Colony formation was reduced to 50% of the controls in LNCaP afer incubation with genistein 0.01 μM (p = 0.004). Higher genistein concentrations (0.1 μM and 10 μM) did not further suppress clonogenic survival. In PC-3 incubation with genistein 0.1 μM decreased colony formation to 75% of the controls (p = 0.027), higher concentrations reduced clonogenic survival further (10 μM: p < 0.001; 25 μM: p < 0.001).

**Figure 3:**
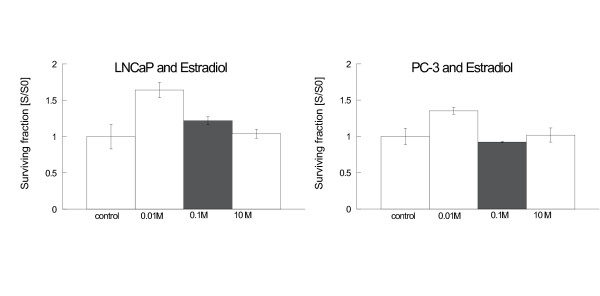
**Clonogenic survival LNCaP (left side) and PC-3 (right side) after incubation with estradiol (LNCaP 48 h incubation, PC-3 24 h)**. Survival was expressed relative to untreated controls. Error bars represent standard errors. In both cell lines estradiol 0.01 μM increased colony formation significantly (LNCaP: p < 0.0001; PC-3: p < 0.0001), while higher concentrations of estradiol did not influence colony formation in comparison to untreated controls.

Incubation of LNCaP and PC-3 with genistein without irradiation resulted in a significant reduction in clonogenic survival in both cell lines (figure 2). In PC-3 cells, this effect appeared to be dose-dependent. In contrast, incubation with estradiol exhibited in low concentrations (0.01 μM) stimulatory effects on the clonogenic survival of both cell lines, while higher concentrations did not alter colony formation ability as compared to controls (figure 3).

### Genistein or estradiol sensitize LNCaP to low radiation doses

**Figure 4:**
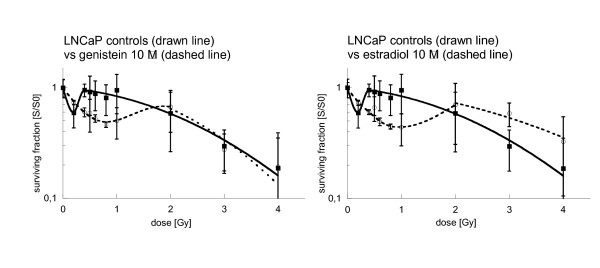
**Survival of LNCaP cells after 24 h pretreatment with genistein 10 μM (left) and with estradiol 10 μM (right), followed by irradiation with single doses between 0.5 and 4 Gy, and by further 24 h of hormonal incubation**. Survival was expressed relative to sham-irradiated controls. Error bars represent standard errors. A polynominal equation was used to fit the low-dose hyper-radiosensitivity region of all curves. Incubation with genistein 10 μM and estradiol 10 μM enlarged the area of radiohypersensitivity to doses of up to 1 Gy when compared to untreated controls. p-values for LNCaP control vs. genistein 10 μM were p < 0.05 at the following dose points: 0.4 Gy, 0.5 Gy, 0.6 Gy, 0.8 Gy, 1 Gy. No significant differences were found between the clonogenic survival curves at 0 Gy, 0.2 Gy, 2 Gy, 3 Gy and 4 Gy. p-values for LNCaP controls vs. estradiol 10 μM were p < 0.05 at the following dose points: 0.4 Gy, 0.6 Gy, 0.8 Gy, 1 Gy and 3 Gy. No significant differences were found at 0 Gy, 0.5 Gy, 2 Gy and 4 Gy.

Clonogenic survival of irradiated LNCaP cells without hormonal incubation did not follow the linear-quadratic model. Instead, a marked hypersensitivity of the cells to low irradiation doses (<0.1 Gy – 0.3 Gy) was revealed (figure 4). Radiation with 0.2 Gy decreased colony formation to 60% compared to unirradiated controls. Higher radiation doses led to a sharp increase in radioresistance: 0.4 Gy reduced clonogenic survival to 95%. This effect has been described before as ”low-dose hyper-radiosensitivity“ [HRS] [30].

However, when cells were incubated with estradiol 10 μM or genistein 10 μM before irradiation, the sensitivity to radiation doses between 0.4 and 2 Gy was significantly increased compared to irradiation alone controls (figure 4). Combination of estradiol incubation and irradiation with 1 Gy resulted in a reduction of clonogenic survival to 45%, after incubation with genistein to 50%. With higher radiation doses (2 Gy or higher) hormonal incubation did not alter clonogenic survival of LNCaP cells significantly compared to controls.

### Genistein and estradiol enhance radioresistance of PC-3 to low radiation doses

**Figure 5:**
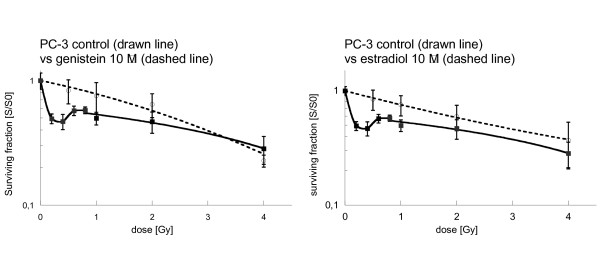
**Survival of PC-3 cells after 24 h pretreatment with genistein 10 μM (left) and with estradiol 10 μM (right), followed by irradiation with single doses between 0.5 and 4 Gy, followed by immediate-plating**. Survival was expressed relative to sham-irradiated controls. Error bars represent standard errors. A polynominal equation was used to fit the low-dose hyper-radiosensitivity region of the control curves while the genistein and estradiol curves followed a linear-quadratic equation. Incubation with genistein and estradiol abolished the HRS to low irradiation doses seen in the controls (genistein 10 μM vs. controls: 0.4 Gy: p < 0.01; 0.6 Gy: p = 0.027; estradiol 10 μM vs. controls: 0.4 Gy: p < 0.001; 0.6 Gy: p < 0.001). At and above 2 Gy there were no significant differences between the surviving clones after hormonal incubation and controls.

Irradiation of PC-3 cells without hormonal incubation revealed a marked HRS to low radiation doses (figure 5). Radiation with 0.3 Gy decreased colony formation to 57% compared to unirradiated controls. With higher radiation doses (0.5 Gy – 1 Gy) radiosensitivity did not increase further, while at 4 Gy clonogenic survival was about 28%.

In contrast to the results obtained with LNCaP, incubation with estradiol 10 μM or genistein 10 μM increased resistance to low irradiation doses in PC-3. Clonogenic survival was significantly higher after hormonal incubation when compared to radiation alone at 1 Gy. The survival curves after hormonal incubation followed the linear-quadratic model. At higher irradiation doses, we did not find a significant difference between hormonal incubation and control.

### Estrogenic stimulation inhibits p21-induction after irradiation in LNCaP

On protein level the expression of p21 was analyzed, as these proteins are involved in cell cycle control. To control for effects of serum withdrawal, controls without serum-withdrawal were investigated, too. Because of mutation of p53 in PC-3 cells, we could not detect any expression of p21 in this cell line [31] (plots not shown).

**Figure 6:**
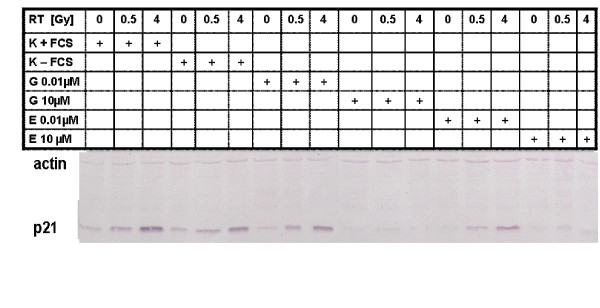
**Western-Blot analysis of p21 and actin in LNCaP after 24 h of hormonal incubation, irradiation with 0.5 Gy and 4 Gy**. 6 h after irradiation the cells were harvested and subjected to protein extraction. The shown results represent one of three assays. In controls incubated in FCS containing media and in controls incubated in media with FCS-withdrawal there was a induction of p21 expression. The same was seen in cells after incubation with low doses of estradiol (0.01 μM) and genistein (0.01 μM). After incubation with high concentrations of estradiol (10 μM) and genistein (10 μM) the induction of p21 was completely abolished.

p21 expression was increased in LNCaP 6 h after irradiation in a radiation-dose dependent manner in controls and after incubation with low concentration of genistein or estradiol (0.01 μM) (figure 6). In contrast, incubation with high hormone concentrations (10 μM) abolished the increase in p21 expression.

### Irradiation increases fraction of cells in G2/M in PC-3

Analysis of cell cycle distribution did not show significant differences in LNCaP cells incubated with estradiol (10 μM) or genistein (10 μM) before irradiation (0.5 Gy, 4 Gy) when compared to controls (serum withdrawal). A high proportion of these cells rested in G0/G1 (about 70%), this proportion was not significantly reduced by hormonal stimulation (data not shown).

**Figure 7:**
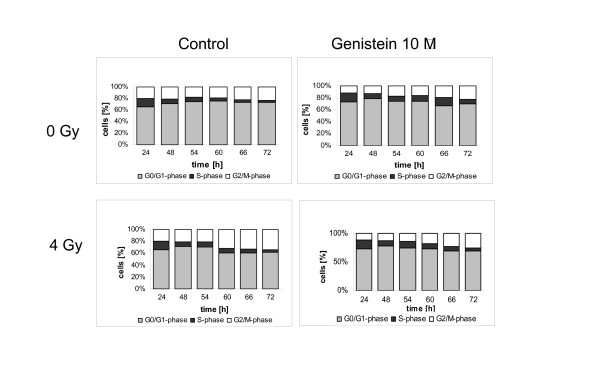
**Cell cycle distribution of PC-3 cells**. At point "0 h" serum was withdrawn. At point "24 h" incubation genistein 10 μM was added to designated probes. At point "48 h" designated probes were irradiated with 4 Gy. In the following time, every 6 h probes were stained with DAPI and analyzed in a flow cytometer up to point "72 h". Every result reflects 3 independent assays. Proportion of cells in G0/G1 is symbolised by a bar, G2/M by a white bar and S-phase by a black bar. In the controls 15% of the cells were in S-phase, 65% in G0/G1 and 20% in G2/M. In the following, the S-phase was reduced to 5%, while G0/G1 increased to 73%. The proportion of cells in G2/M showed minimal changes. After incubation with genistein 10 μM no significant differences when compared to untreated controls were observed. After irradiation with 4 Gy the proportion of cells in G0/G1 decreased from 73% to 62%, in S-phase decreased from 8% to 3.6% and in G2/M phase increased from 21% to 34.3%. Similar results were observed after incubation with genistein 10 μM and irradiation with 4 Gy. In the controls 15% of the cells were in S-phase, 65% in G0/G1 and 20% in G2/M. In the following, the S-phase was reduced to 5%, while G0/G1 increased to 73%. The proportion of cells in G2/M was only minimal changes. After incubation with genistein 10 μM no significant differences when compared to untreated controls were observed. After irradiation with 4 Gy the proportion of cells in G0/G1 decreased from 73% to 62%, in S-phase decreased from 8% to 3.6% and in G2/M phase increased from 21% to 34.3%. Similar results were observed after incubation with genistein 10 μM and irradiation with 4 Gy.

In PC-3 cells unirradiated controls exhibited a nearly constant G2/M fraction during the whole time course (about 20%, figure 7). However, the S-phase fraction decreased from 15% at the beginning of the observation (24 h after serum withdrawal) to 5% 66 h after serum withdrawal. Comparable cell cycle distribution characteristics were seen after incubation with 10 μM genistein. Only 66 h after serum withdrawal a higher proportion of the cells in S-phase were detected.

In contrast, irradiation (4 Gy) of PC-3 cells after serum withdrawal resulted in a significant increase in the fraction of cells in G2/M (from 21% before irradiation to 34% 12 h after irradiation [timepoint 60 h, figure 7]). At the same time-course a reduction of cells in G1 from 73% before irradiation to 62% 12 h after irradiation was noticed.

Interestingly, incubation with genistein 10 μM reduced the amount of cells in G2/M after 4 Gy irradiation when compared to irradiation alone (figure 7). Comparable results were achieved with estradiol incubation and irradiation (data not shown).

Irradiation with 0.5 Gy had no significant influence on cell cycle distribution neither after serum withdrawal nor after hormone incubation (data not shown).

## Discussion

For easier reading the results of the study are summarized in table 1.

**Table 1:** Summary of the results

	***cell line***	***treatment***	***results***
receptor expression	LNCaP		ER-α + ER-β +
	PC-3		ER-α 0 ER-β 0

clonogenic survival	LNCaP	genistein	survival reduced
		estradiol	survival increased (0.01 μM)/no effect (higher concentrations)
		genistein + RT	area of HRS enlarged
		estradiol + RT	area of HRS enlarged
	PC-3	genistein	survival reduced
		estradiol	survival increased (0.01 μM)/no effect (higher concentrations)
		genistein + RT	HRS abolished
		estradiol + RT	HRS abolished

p21 expression	LNCaP	RT	increased expression
		RT + genistein 10 μM	reduced expression
		RT + estradiol 10 μM	reduced expression
	PC-3		no expression

FACS (cell cycle)	LNCaP	controls	G0/G1 arrest
		genistein or estradiol	G0/G1 arrest
		RT	G0/G1 arrest
	PC-3	genistein or estradiol	no influence
		RT	G2/M arrest
		RT + genistein or estradiol	G2/M arrest reduced

Our passages of LNCaP cells stained positive for ER-α and ER-β, but no expression of ER-α or ER-β was seen in the investigated PC-3 passages. RNA expression of these receptors in LNCaP has been shown before [3,4]. However, other groups reported contradictory results [32,33]. In PC-3 cells RNA expression of ER-β has been reported [3]. Others found PC-3 cells to be positive for both ER types [4,32]. These differing results are explained by differences in the passages of the studied cell lines, previous and present growth conditions, and in the applied methodologies.

In the interpretation of our data on clonogenic survival we have to take different incubation times for both cell lines into account. Due to methodological problems (slow growing LNCaP cells – see materials and methods), LNCaP were incubated for 48 h and PC-3 for 24 h with genistein or estradiol. We do not feel that these protocol variations may sufficiently explain the differences observed in clonogenic survival between both cell lines.

### Effects of hormone incubation

Incubation with genistein for 24 h – 48 h reduced clonogenic survival in both studied cell lines. Similar results have been reported by other groups [16,18,34,35]. Taken together, the effects of genistein on survival in these cell lines seem to be independent of ER- and p53-expression.

In contrast, low concentrations of estradiol (0.01 μM) stimulated clonogenic growth in both cell lines, while higher concentrations did not exhibit a significant effect in comparison to controls. In LNCaP cells, these results are supported by proliferation studies where cells were incubated up to 5 days with concentrations between 0.0001 – 10 μM [36,37]. In these studies even high estradiol concentrations induced cell proliferation. In contrast to our results, other studies (using serum-containing media and other endpoints than clonogenic survival) reported reduction of cell proliferation in PC-3 after incubation with 0.1 μM estradiol [38].

### Effects of irradiation alone

HRS of LNCaP and PC-3 cells to low irradiation doses (<0.1 Gy – 0.3 Gy) has been described before [30]. The cells are very sensitive to small single radiation doses but become more resistant to larger single doses (at about 1 Gy). Explanations for this phenomen have been proposed by Marples et al. in regard to damage recognition, signal transduction and damage repair [39]. Amongst others they postulate a rapidly occurring dose-dependent pre-mitotic cell cycle checkpoint that is specific to cells irradiated in the G2-phase. The activation of this checkpoint seems to be dependent on a threshold dose. However, the clinical relevance of HRS is debatable. To our knowledge, up to now HRS effects were only described *in vitro*. An *in vivo *proof has not been published yet.

Our data support an independence of the HRS in regard to ER- or p53/p21-expression.

### Effects of the combination of irradiation and hormone incubation

In combination with irradiation both tested hormones exhibited similar effects on clonogenic survival dependent on the investigated cell line. In LNCaP, incubation with genistein as well as estradiol increased the area of HRS (including the 1 Gy dose point). To our knowledge, such effect has not been reported before.

In PC-3 we found a completely different effect as hormonal incubation abolished the HRS observed in the irradiated controls by increasing radioresistance. Clonogenic survival was best described with the linear-quadratic model also at low irradiation dose points.

Hillman et al. investigated the combination between irradiation and genistein incubation (5–30 μM, 24 h before irradiation – 10 d after irradiation) on clonogenic survival of PC-3 cells, too [24]. Only a concentration of 15 μM genistein reduced clonogenic survival at all measured irradiation doses, lower genistein doses had no effect. Survival curves followed the linear-quadratic model, too. They did not describe HRS to low irradiation doses, as only doses of 2 Gy and higher (photon beam) were evaluated. Further methodological differences to our study were the use of FCS-containing media during genistein incubation and the incubation with higher genistein doses.

### Mechanisms of interaction

To search for mechanisms of interaction we investigated protein expression of cell cycle controlling proteins. As PC-3 cells expressed not functional p53 we could not detect p21 expression in these cells. In LNCaP p21 expression was increased as a downstream signal transduction protein of p53 after irradiation when compared to controls. Incubation with high concentrations of genistein or estradiol abolished this p21 expression after irradiation. In unirradiated controls no p21 expression was detectable.

These data are in contrast to the literature. Shen et al. showed a dose-dependent increase in p21 expression after incubation with genistein (without irradiation, 0 – 80 μM for 24 h) [17]. Similar results were obtained by another study after incubation with 5 μM for 6 h – 12 h [40].

With FACS-analysis we tried to verify our Western Blot results in terms of cell cycle regulation. However, as our passages of LNCaP cells proliferated very slowly in FCS-free medium (time for cell doubling 5 days), the majority of cells was in G0/G1. Incubation with hormones did not dissolve this accumulation. With such a high level of cells in G0/G1 in control cells, the increase after irradiation in this proportion of cells did not reach significance. Therefore, short term effects as seen in western blotting did not result in significant changes in cell cycle distribution. Effects described in clonogenic survival were not explained by the results of cell cycle analysis.

In PC-3 cells, incubation with estradiol 10 μM or genistein 10 μM did not alter cell cycle distribution significantly when compared to controls. However, irradiation with 4 Gy induced a G2/M cell cycle arrest, but not irradiation with 0.5 Gy. This result is explained by the missing of functional p53, thus lacking of any G0/G1 arrest. The G2/M arrest after irradiation with high doses was not abolished by estrogenic stimulation. These results are supported by another study that reported a G2/M cell cycle arrest 72 h and 96 h after irradiation with 3 Gy or after incubation with genistein concentrations of 15 – 30 μM in FCS-containing media [41]. In this study the NF-κB activity was investigated, too. An inhibition of radiation-induced activation of NF-κB activity by genistein pretreatment could be shown. Furthermore, a significant increase in cleaved PARP protein was measured following combined genistein and radiation treatment, indicating increased apoptosis. The authors proposed a mechanism of increased cell death by genistein and radiation via inhibition of NF-κB, leading to altered expression of regulatory cell cycle proteins, thus promoting G2/M arrest and increased radiosensitivity [41]. However, as it is doubtful whether apoptosis is clinical relevant in irradiated solid tumor cells, we did not measure this endpoint in our study [42].

One potential interaction between estrogenic stimulation and irradiation could be identified in ER-α and ER-β positive LNCaP cells. Irradiation induces double strand breaks, these are recognized and via phosphorylation of ATM, p53 and p21 a G0/G1 arrest is induced. Activated ERs interfere with this cascade by inducing degradation of p21, thus abolishing G0/G1 arrest [43]. However, these cascades do not explain the increased area of HRS seen in clonogenic survival analysis after incubation with genistein or estradiol. Furthermore, we could not identify the molecular mechanism of the results observed in ER-α and ER-β negative PC-3 cells.

Taken together, since we showed comparable effects of genistein and estradiol in combination with irradiation in both studied cell lines neither ER- nor p53-expression seemed to play a role in the linked signalling. Nevertheless, both compounds targeted the same molecular switch, that we were not able to identify.

## Conclusion

We observed an increased HRS to low irradiation doses after incubation with estradiol 10 μM and genistein 10 μM in ER-α and ER-β positive LNCaP cells. In contrast, in ER-α and ER-β negative PC-3 cells, we observed an abolishing of the HRS to low irradiation doses by hormonal stimulation. In conclusion, HRS was independent from ER- or p53/p21-expression. It was modulated by genistein and estradiol dependent from the genetic background of the investigated cell line. Furthermore, the effects of both tested compounds on survival were ER and p53 independent. Since genistein and estradiol effects in both cell lines were comparable, neither ER- nor p53-expression seemed to play a role in the linked signalling. Nevertheless both compounds targeted the same molecular switch. To identify the underlying molecular mechanisms, further studies are needed.

The observation of an extended HRS of PC cells after incubation with genistein or estradiol would be of high clinical interest, especially as LNCaP reflects a locally advanced, androgen dependent PC. This would mean, that PC could be irradiated with decreased irradiation doses, resulting in reduced normal tissue toxicity. However, as our data are based on *in vitro *observations only, these results have to be interpreted with caution. To our knowledge, no *in vivo *proof for HRS to low irradiation doses has been published up to day.

## Competing interests

The authors declare that they have no competing interests.

## Authors' contributions

RMH outlined the study, helped HAW to perform the majority of the experimental work and drafted the manuscript. MRF supervised the radiobiological experiments and molecularbiological work. PT carried out the cell cycle analyses. HJ participated in the planning of the experiments and the Western Blot analyses. HS conceived the study and helped with coordination. CG performed immunostaining. HC participated in its design and coordination and helped to draft the manuscript.

All authors read and approved the final manuscript.
